# Chronic pain among public safety personnel in Canada

**DOI:** 10.1080/24740527.2017.1410431

**Published:** 2017-12-18

**Authors:** R. N. Carleton, T. O. Afifi, S. Turner, T. Taillieu, R. El-Gabalawy, J. Sareen, G. J. G. Asmundson

**Affiliations:** a Anxiety and Illness Behaviours Laboratory, Department of Psychology, University of Regina, Regina, Saskatchewan, Canada; b Rady Faculty of Health Sciences, University of Manitoba, Winnipeg, Manitoba, Canada

**Keywords:** chronic pain, first responders, public safety personnel, work injuries

## Abstract

**Background**: Chronic pain is highly prevalent in the general population and may be even higher among public safety personnel (PSP; e.g., correctional officers, dispatchers, firefighters, paramedics, police). Comprehensive data on chronic pain among diverse Canadian PSP are relatively sparse.

**Aims**: The current study was designed to provide initial estimates of chronic pain frequency and severity among Canadian PSP.

**Methods**: Estimates of chronic pain frequency and severity (i.e., intensity and duration) at different bodily locations were derived from self-reported data collected through an online survey. Participants included 5093 PSP (32.5% women) grouped into six categories (i.e., call center operators/dispatchers, correctional officers, firefighters, municipal/provincial police, paramedics, Royal Canadian Mounted Police [RCMP]).

**Results**: Substantial proportions of participants reported chronic pain, with estimates ranging from 35.3% to 45.4% across the diverse PSP categories. Across PSP categories, chronic lower back pain was the most prevalent. For some pain locations, firefighters and municipal/provincial police reported lower prevalence, but paramedics reported lower intensity, and duration, than some other PSP groups. Over 50% of RCMP and paramedics reporting chronic pain indicated that the pain was associated with an injury related to active duty.

**Conclusions**: Discrepancies emerged across PSP members with respect to prevalence, location, and severity. The current data suggest that additional resources and research are necessary to mitigate the development and maintenance of distressing or disabling chronic pain for Canadian PSP.

## RÉSUMÉ

**Contexte** : La prévalence de la douleur chronique est élevée parmi la population en général et pourrait être encore plus élevée chez le personnel de la sécurité publique (ex.: agents correctionnels, répartiteurs, pompiers, ambulanciers, policiers). Il y a relativement peu de données exhaustives sur la douleur chronique chez le personnel de sécurité publique canadien.

**But** : Cette étude visait à estimer la fréquence et la gravité de la douleur chronique chez le personnel de sécurité publique canadien.

**Méthodes** : Des estimations de la fréquence et de la gravité de la douleur chronique (i.e. intensité et durée) à différents endroits du corps ont été tirées de données autodéclarées receuillies par le truchement d’un questionnaire en ligne. Les 5 093 participants (32,5 % de femmes) ont été regroupés en six catégories (i.e. opérateurs et répartiteurs en centre d’appel, agents correctionnels, pompiers, policiers au niveau municipal ou provincial, ambulanciers, Gendarmerie royale du Canada.)

**Résultats** : Une proportion importante de participants a déclaré souffrir de douleur chronique, avec des estimations allant de 35,3 % à 45,4 % pour toutes les catégories de personnel de sécurité. La douleur au bas du dos présentait la prévalence la plus élevée dans toutes les catégories de personnel de sécurité. Les ambulanciers ont déclaré une plus faible prévalence de l’intensité et de la durée de la douleur à ressentie à certains endroits, comparativement aux autres groupes de personnel de la santé publique. Plus de 50 % des agents de la Gendarmerie royale du Canada et des ambulanciers qui ont déclaré souffrir de douleur chronique ont affirmé que la douleur était associée à une blessure subie pendant qu’ils étaient en service.

**Conclusions** : Des différences ont été relevées entre les membres du personnel de la sécurité publique en ce qui concerne la prévalence, la localisation et la gravité de la douleur. Les données actuelles suggèrent que des ressources et des études additionnelles sont nécessaires pour atténuer l’apparition et la persistance de la douleur chronique éprouvante ou invalidante chez le personnel de la sécurité publique canadien.

## Introduction

Chronic pain is characterized by pain lasting longer than the typical 3-month duration for healing damaged tissue.^1^ The most prevalent chronic pain locations are the lower back, neck, upper extremities, and head.^2^ Chronic pain impacts a substantial proportion of the Canadian general population,^3^ with approximately one in four persons reporting chronic pain in the past month.^4–6^ Women appear more likely to report chronic pain than men and chronic pain rates appear to increase with age.^7^ The annual cost of chronic pain in Canada is estimated to exceed 6 billion dollars.^2^

Canadian public safety personnel (PSP) include persons working as correctional workers (security and non-security roles), dispatchers, firefighters, paramedics, and police officers.^8^ PSP work typically involves regular periods of substantial physical stress (e.g., engaging with public safety incidents such as fires, resuscitations, arrests) as well as extended periods of potential inactivity (e.g., time between duty calls).^9^ Exposure to potentially traumatic events (e.g., exposure to threatened or actual physical assaults, fires, or explosions^10^) is considered common for persons working as PSP,^11,12^ and researchers have substantiated an important link between trauma and chronic pain in several other populations (e.g., military).^13–17^ Despite the costs of chronic pain, the potential physical stressors, and the potentially problematic links between trauma and pain, the available information on chronic pain among PSP remains sparse, particularly in Canada.^9^ Further, the extant data are typically focused on a single PSP category as opposed to a range of personnel, and the assessments used diverse data collection methods (e.g., broad sampling vs. sampling of injured persons only), tools (e.g., records review, self-report), sample sizes, pain questions, and time frames.

With respect to the limited available Canadian data, previous estimates from a large sample of Royal Canadian Mounted Police (RCMP; *n *= 1002) placed lifetime and past-year chronic pain prevalence at 54.9% and 41.8%, respectively, with most participants who reported chronic pain (91.5%) reporting that the pain started after joining the service^18^; however, the cross-sectional nature and limited scope of the data preclude firm conclusions regarding temporal sequencing and exclusion of cofounds (e.g., age). A study of Canadian paramedics (*n *= 101) indicated that 88% reported having “musculoskeletal problems (pain, aches, or discomfort)” (p. 23) but not necessarily chronic pain.^19^ In a sample of Canadian firefighters (*n *= 294), the prevalence of self-reported musculoskeletal disorder ranged from 20% to 45%, depending on the affected bodily area identified as problematic by participants (e.g., neck, lower extremity, arm, shoulder, hand); however, the researchers did not specifically report on chronic pain.^20^

There have also been international efforts to estimate the prevalence of chronic pain among PSP. Research with Irish correctional officers showed that nearly half report chronic pain.^21^ Researchers assessing chronic back pain in two large European samples of police (i.e., Northern Ireland, *n *= 2000; Manchester, *n *= 600) estimated the prevalence as ranging from 4.3% to 8.2%.^22^ A study using a smaller sample of U.K. police (*n *= 80) found that chronic pain prevalence estimates ranged from 22% to 38%.^23^ Only 10% of Swiss paramedics (*n *= 334) reported chronic pain during the past 12 months^24^; similarly, in a large sample of randomly selected Swedish paramedics (*n *= 1500), 10% reported pain that limited their activities during the past 12 months.^25^ In contrast, approximately half (50.5%) of a large American paramedic sample (*n *= 930) reported having pain for one or more days in a 2-week period.^26^ In an American sample of urban firefighters (*n *= 382), approximately 13% reported pain occurring often or frequently within the prior week.^27^ Finally, a qualitative study of 17 American 911 dispatchers evidenced reports of somatic concerns, including chronic pain, as being common among dispatchers.^28^ Based on the available, albeit sparse, literature, there appear to be differences in pain reporting based on PSP population and country; accordingly, data derived from Canadian PSP are needed.

The available Canadian and international data indicate substantial variability in pain estimates and appear insufficient with respect to understanding the prevalence of chronic pain in PSP. There appears to be no Canadian data for PSP working as correctional workers, dispatchers, or municipal/provincial police officers. The apparent variability in results raises questions regarding chronic pain estimate reliability and precludes comparisons among PSP groups. The objectives of the current study were to (1) provide initial estimates of current self-reported rates of chronic pain frequency, intensity, and duration at different bodily locations across a diverse and large sample of Canadian PSP and (2) assess the perceived precipitants of PSP chronic pain, with work-related mechanisms of injury expected to be the primary perceived cause.

## Materials and methods

### Procedure

The current data were collected using a secured web-based self-report survey made available to PSP participants in English or French as part of a larger study.^29^ The research followed established guidelines for web-based surveys.^30^ The survey included well-established measures for screening chronic pain (details below). Authors and representatives from the Public Safety Steering Committee (PSSC) of the Canadian Institute for Public Safety Research and Treatment (CIPSRT) selected measures using a collaborative approach. Details of the PSSC, CIPSRT, and survey procedures are available elsewhere.^29^ The study was approved by the University of Regina Institutional Research Ethics Board (File #2016–107). Participants could access the survey from September 1, 2016, to March 31, 2017. The survey issued each participant a unique computer-generated random code that allowed for repeated nonduplicate entries, therein accommodating PSP schedules and facilitating participation.

Participation was solicited through e-mails sent to actively working PSP, including civilian members working for police and volunteer firefighters. The e-mails were sent by the CIPSRT PSSC. The PSSC includes leadership representatives from each of the national associations for PSP. The minister of public safety and emergency preparedness also provided a video invitation encouraging participation. Each of the PSSC member agencies sent the invitation e-mail to their provincial counterparts for forwarding either directly to potential participants or to their municipal counterparts for forwarding to invite potential participants. Several advocacy organizations also sent the invitation to their e-mail distribution lists. Many social media outlets also made the invitation available. Accordingly, the invitation process prohibits accurate estimations of how many persons were invited to participate. Additional data collection details are reported elsewhere.^29^

A total of *N *= 8520 began the survey and answered at least the first question (i.e., “Please indicate which category of First Responders or other Public Safety Personnel you feel best describes your current occupation”). For the current study, only PSP who reported working as call center operators/dispatchers, correctional officers, firefighters, municipal/provincial police, paramedics, or RCMP were included because the sample sizes were considered large enough to provide defensible estimates; however, only *N *= 5093 (59.8%) persons progressed far enough through the survey to be asked about the presence of chronic pain and then completed the associated sections required to be included in the current analyses.

### Chronic pain

Current chronic pain experiences were assessed with items based on work done by the International Association for the Study of Pain^31^ and previous reviews^32–36^; specifically, we asked participants: “Do you experience chronic pain defined by pain more days than not, lasting longer than 3 months?” Response options included “Yes” (*n *= 1859), “Yes, but I’d rather not discuss it” (*n *= 176), “No” (*n *= 3030), and “Prefer not to answer” (*n *= 28). Participants who responded to the pain question with yes were subsequently asked about location (see Table 2), intensity (0 = *no pain*; 10 = *pain that sends you to the hospital*), and duration (How many days per week, on average? For how many months? see Table 3). Participants who identified more than one location were also asked to select the location that causes the most interference (“If you indicated that you have multiple locations of chronic pain, which interferes most with your life?”). Participants were subsequently asked about the cause of the chronic pain that most interfered with their life (see Table 4). The variable for perceived cause of chronic pain (“What caused the chronic pain that most interferes with your life?”) was not mutually exclusive (i.e., instructions were to check all that apply).

**Table 1.** Demographic characteristics among PSP in Canada, *N* = 5093.^a^

	% (*n*)
Sex	
Male	67.5 (3425)
Female	32.5 (1650)
Age	
19–29	7.5 (379)
30–39	28.0 (1419)
40–49	36.7 (1858)
50–59	24.5 (1241)
60 and older	3.3 (168)
Marital status	
Married/common-law	75.9 (3835)
Single	10.1 (510)
Separated/divorced/widowed	10.5 (529)
Remarried	3.5 (179)
Ethnicity	
White	91.6 (4615)
Other	8.4 (423)
Education	
High school or less	9.2 (455)
Some postsecondary (less than 4-year college/university program)	54.2 (2694)
University degree/4-year college or higher	36.6 (1821)
Province of residence	
Western Canada (BC, AB, SK, MB)	52.4 (2645)
Eastern Canada (ON, QC)	34.3 (1732)
Atlantic Canada (PEI, NS, NB, NFL)	12.0 (607)
Northern Territories (YK, NWT, NVT)	1.2 (61)
Urban/rural work location	
Urban	93.9 (4663)
Rural	6.1 (303)
Years of service	
Less than 4 years	4.9 (245)
4 to 9 years	17.4 (876)
10 to 15 years	23.3 (1174)
More than 15 years	54.4 (2743)
Public safety officer category	
Municipal/provincial police	26.5 (1348)
RCMP	25.6 (1305)
Corrections workers	13.9 (710)
Firefighters	15.8 (807)
Paramedics	13.3 (675)
Call center operators/dispatchers	4.9 (248)

^a^PSP indicates public safety personnel; RCMP, Royal Canadian Mounted Police. *N* for each variable differ slightly due to nonresponse.

**Table 2.** Prevalence and location of chronic pain in total sample and by PSP category.^a^

	Total sample	Municipal/provincial police^1^	RCMP^2^	Corrections workers^3^	Firefighters^4^	Paramedics^5^	Call center operators/dispatchers^6^	Chi-square	Significant differences between PSP categories
Prevalence, % (*n*)									
Any chronic pain	40.2 (2035)	35.9 (482)	43.4 (563)	45.4 (318)	35.3 (284)	44.1 (297)	36.7 (91)	37.099***	1 < 2, 3, 5
									4 < 2, 3, 5
									6 < 2, 3, 5
Chronic pain location, % (*n*)^b,c^									
Lower back	24.0 (1216)	22.1 (296)	26.5 (344)	26.1 (183)	18.4 (148)	28.9 (195)	20.2 (50)	33.845***	1 < 2, 3, 5
									4 < 1, 2, 3, 4
									6 < 2, 5
Shoulder	17.6 (890)	14.7 (197)	19.6 (254)	18.5 (130)	15.7 (126)	21.5 (145)	15.3 (38)	21.985***	1 < 2, 3, 5
									4 < 2, 5
									6 < 5
Neck	16.8 (849)	15.4 (207)	17.8 (231)	21.0 (147)	12.3 (99)	18.1 (122)	17.3 (43)	23.993***	1 < 3
									4 < 1, 2, 3, 5, 6
Arm	11.1 (563)	10.6 (142)	12.5 (162)	12.6 (88)	6.7 (54)	12.5 (84)	13.3 (33)	22.561***	4 < 1, 2, 3, 5, 6
Leg	14.3 (726)	12.7 (171)	15.7 (204)	16.5 (116)	11.3 (91)	15.0 (101)	17.3 (43)	15.666**	1 < 2, 3
									4 < 2, 3, 5, 6
Hand	10.9 (554)	9.8 (131)	12.0 (156)	13.6 (95)	7.2 (58)	12.0 (81)	13.3 (33)	22.112***	1 < 3
									4 < 1, 2, 3, 5, 6
Foot	12.6 (637)	11.8 (159)	14.9 (193)	15.4 (108)	7.6 (61)	12.2 (82)	13.7 (34)	30.671***	1 < 2, 3
									4 < 1, 2, 3, 5, 6
Headaches	15.2 (769)	14.4 (193)	16.0 (207)	18.5 (130)	9.1 (73)	18.8 (127)	15.7 (39)	37.774***	1 < 3, 5
									4 < 1, 2, 3, 5, 6
Other	7.1 (358)	5.7 (77)	8.8 (114)	6.8 (48)	5.2 (42)	9.1 (61)	6.5 (16)	17.900**	1 < 2, 5
									4 < 2, 5

^a^PSP indicates public safety personnel; RCMP, Royal Canadian Mounted Police.

^b^Nonmutually exclusive chronic pain locations.

^c^Only calculated for respondents who reported experiencing any chronic pain, more days than not, that lasted longer than 3 months.

Different numbered superscripts indicate that public safety personnel categories differ from one another at 0.05 only. Differences across categories were tested using logistic regression models for prevalence. *P ≤ .05; **P ≤ .01; ***P ≤ .001

**Table 3.** Intensity and duration of chronic pain in total sample and by PSP category.^a^

	Total sample	Municipal/provincial police^1^	RCMP^2^	Corrections workers^3^	Firefighters^4^	Paramedics^5^	Call center operators/dispatchers^6^	*F*-value	Significant difference between PSP categories
Intensity, mean (SD)^b,c^ score range 0 to 10									
Lower back	4.41 (2.24)	4.65 (2.30)	4.37 (2.19)	4.77 (2.28)	4.10 (2.14)	4.07 (2.27)	4.22 (1.98)	3.210**	5 < 3
Shoulder	3.64 (2.49)	3.42 (2.70)	3.94 (2.27)	3.81 (2.73)	3.83 (2.32)	2.82 (2.23)	4.61 (2.63)	5.762***	5 < 2, 3, 4, 6
Neck	3.72 (2.57)	3.74 (2.77)	3.65 (2.56)	4.36 (2.49)	3.34 (2.57)	3.18 (2.33)	4.14 (2.20)	3.654**	4 < 3; 5 < 3
Arm	1.94 (2.52)	1.78 (2.41)	1.94 (2.60)	2.24 (2.67)	2.40 (2.65)	1.29 (2.08)	2.64 (2.71)	2.385*	NS
Leg	3.05 (2.77)	3.16 (2.81)	3.15 (2.78)	3.24 (3.03)	3.34 (2.60)	2.27 (2.43)	2.86 (2.78)	2.052 (NS)	NS
Hand	1.72 (2.29)	1.64 (2.33)	1.71 (2.24)	2.29 (2.70)	1.76 (2.11)	1.09 (1.61)	1.88 (2.46)	2.549*	5 < 3
Foot	2.31 (2.68)	2.38 (2.78)	2.42 (2.60)	2.69 (2.94)	2.30 (2.39)	1.62 (2.36)	1.91 (2.77)	1.760 (NS)	NS
Headaches	3.98 (3.16)	4.21 (3.28)	3.83 (2.97)	4.52 (3.21)	3.34 (3.10)	3.65 (3.17)	4.00 (3.30)	1.949 (NS)	NS
Other	3.89 (2.75)	3.61 (3.01)	3.78 (2.71)	4.10 (3.03)	3.88 (2.28)	4.03 (2.50)	4.94 (2.91)	0.750 (NS)	NS
Duration, mean (SD)^b,c^ days per week									
Lower back	5.09 (2.19)	5.16 (2.25)	5.25 (2.14)	5.06 (2.23)	4.83 (2.25)	4.97 (2.10)	4.81 (2.23)	1.005 (NS)	NS
Shoulder	4.68 (2.60)	4.53 (2.78)	5.15 (2.39)	4.72 (2.57)	4.64 (2.46)	4.00 (2.70)	4.53 (2.69)	3.544**	5 < 2
Neck	4.55 (2.56)	4.63 (2.57)	4.62 (2.63)	5.00 (2.31)	4.19 (2.74)	4.12 (2.63)	4.47 (2.17)	1.781 (NS)	NS
Arm	3.03 (3.20)	2.94 (3.15)	3.00 (3.30)	3.61 (3.13)	3.67 (3.13)	2.27 (3.00)	2.83 (3.03)	1.564 (NS)	NS
Leg	4.25 (3.00)	4.62 (2.91)	4.22 (3.00)	4.52 (3.12)	4.26 (2.88)	3.38 (3.01)	4.32 (3.07)	2.016 (NS)	5 < 1
Hand	2.89 (3.09)	2.81 (3.06)	2.89 (3.20)	3.44 (3.11)	3.14 (3.18)	2.29 (2.93)	2.67 (2.70)	1.043 (NS)	NS
Foot	3.46 (3.22)	3.67 (3.20)	3.68 (3.28)	3.72 (3.19)	2.90 (3.10)	2.88 (3.14)	2.85 (3.27)	1.291 (NS)	NS
Headaches	2.42 (2.28)	2.38 (2.32)	2.49 (2.32)	2.86 (2.31)	2.13 (2.36)	2.29 (2.22)	1.69 (1.61)	1.812 (NS)	NS
Other	4.91 (2.65)	5.20 (2.67)	4.96 (2.66)	4.20 (2.83)	5.18 (2.44)	4.92 (2.67)	4.50 (2.50)	0.895 (NS)	NS
Duration, mean (SD)^b,c,d^ total number of months									
Lower back	39.82 (37.87)	40.71 (39.04)	42.03 (38.80)	37.12 (36.29)	33.00 (34.02)	43.03 (39.55)	35.90 (31.79)	1.435 (NS)	NS
Shoulder	29.51 (33.13)	25.64 (32.99)	34.23 (34.15)	30.24 (33.36)	27.93 (33.47)	26.15 (30.78)	33.84 (32.08)	1.643 (NS)	NS
Neck	33.83 (36.05)	32.78 (36.79)	37.45 (37.66)	32.71 (33.48)	30.00 (33.77)	30.34 (36.29)	41.50 (35.38)	1.066 (NS)	NS
Arm	16.29 (28.24)	16.33 (27.32)	16.04 (28.29)	16.15 (25.90)	18.53 (35.55)	15.23 (28.96)	16.40 (23.09)	0.073 (NS)	NS
Leg	27.23 (33.63)	29.49 (35.15)	29.78 (34.69)	27.09 (35.04)	23.34 (27.24)	22.88 (33.04)	25.38 (33.31)	0.782 (NS)	NS
Hand	17.57 (30.19)	16.85 (26.42)	19.95 (34.01)	19.93 (31.34)	17.98 (32.43)	11.57 (27.10)	16.00 (21.93)	0.704 (NS)	NS
Foot	17.39 (26.71)	18.20 (28.04)	20.44 (29.20)	15.34 (22.16)	14.94 (23.76)	14.18 (25.87)	12.60 (22.50)	0.942 (NS)	NS
Headaches	28.53 (35.30)	26.37 (32.13)	30.24 (36.78)	35.41 (37.28)	20.19 (28.95)	27.99 (36.75)	25.45 (38.90)	1.479 (NS)	NS
Other	33.11 (36.09)	26.84 (32.12)	34.87 (38.55)	24.06 (26.80)	41.56 (38.33)	39.96 (38.78)	22.07 (30.88)	1.926 (NS)	NS

^a^PSP indicates public safety personnel; RCMP, Royal Canadian Mounted Police.

^b^Nonmutually exclusive chronic pain locations.

^c^Only calculated for respondents who reported experiencing any chronic pain, more days than not, that lasted longer than 3 months.

^d^To prevent outliers from skewing the mean, duration of months was limited to a range of a minimum of 0 months to a maximum of 150 months. This equated to removing the top 10% or less of the sample for each type of pain to calculate the mean and SD for the duration of months of pain.

Different numbered superscripts indicate that public safety personnel categories differ from one another at 0.05 only. NS = no significant differences between PSP categories. Differences across categories were tested using analyses of variance with Tukey’s honestly significant difference test for mean scores. *P ≤ .05; **P ≤ .01; ***P ≤ .001.

**Table 4.** Perceived cause of chronic pain in total sample and by PSP category.^a^

Perceived cause, % (*n*)^b,c^	Total sample	Municipal/provincial police^1^	RCMP^2^	Corrections workers^3^	Firefighters^4^	Paramedics^5^	Call center operators/dispatchers^6^	Chi-square	Significant difference between PSP categories
Injury related to active duty	40.2 (818)	39.6 (191)	54.4 (306)	24.5 (78)	29.2 (83)	50.5 (150)	11.0 (10)	139.110***	1 < 2, 5
									3 < 1, 2, 5
									4 < 1, 2, 5
									6 < 1, 2, 3, 4, 5
Injury related to work other than active duty	9.6 (195)	10.2 (49)	7.3 (41)	11.6 (37)	11.6 (33)	9.4 (28)	7.7 (7)	6.918 (NS)	2 < 3, 4
Injury not related to work	16.2 (329)	18.9 (91)	8.5 (48)	20.8 (66)	19.4 (55)	14.8 (44)	27.5 (25)	42.937***	2 < 1, 3, 4, 5, 6
									5 < 6
Non-injury-related disease (e.g., osteoarthritis)	11.2 (228)	10.0 (48)	7.8 (44)	14.8 (47)	11.3 (32)	12.1 (36)	23.1 (21)	24.484***	1 < 3, 6
									2 < 3, 5, 6
									4 < 6
									5 < 6

^a^PSP indicates public safety personnel; RCMP, Royal Canadian Mounted Police.

^b^Options for perceived cause of pain are nonmutually exclusive.

^c^Only calculated for respondents who reported experiencing any chronic pain, more days than not, that lasted longer than 3 months.

Different numbered superscripts indicate that public safety personnel categories differ from one another at 0.05 only. NS = no significant differences between PSP categories. Differences across categories were tested using logistic regression models for prevalence estimates. *P ≤ .05; **P ≤ .01; ***P ≤ .001.

### Statistical analyses

To determine the representativeness of the sample, the demographic proportions for sex, age, and provincial region in the current sample were compared to data provided by Statistics Canada for PSP using the 2011 National Household Survey and the National Occupational Classification.^37^ Comparative results indicated that sex and age distributions were similar across PSP groups (see previous publication^29^). All analyses were conducted using SPSS Version 24 software (IBM Corp, Armonk, NY).

First, the overall demographic characteristics of the population were calculated (Table 1). Second, prevalence estimates for any chronic pain and chronic pain location were first calculated across PSP groups using cross tabulations with chi-square tests of association (Table 2). Additionally, means and standard deviations were calculated for severity factors (intensity and duration; see Table 3). Among those endorsing any chronic pain, prevalence estimates are reported for perceived cause. Logistic regression models were computed to test for differences in prevalence estimates across PSP groups for categorical variables. In the logistic regression models, the chronic pain variables were entered as the dependent variable and the PSP groups were entered as the independent variable. Differences across the PSP groups were tested by changing the reference group for the independent variable in the logistic regression models. One-way analyses of variance with Tukey’s post hoc comparisons were conducted to test for differences across PSP groups for continuous variables (Table 3). Statistical significance was determined at *P *≤ 0.05

## Results

### Chronic pain prevalence

The estimated proportion of Canadian PSP reporting chronic pain was 40.2%. Table 2 includes the prevalence of any chronic pain and chronic pain location across PSP categories, as well as results from the logistic regression models to test differences between PSP categories. Chronic pain estimates range from 35.3% (firefighters) to 45.4% (correctional workers). The highest prevalence of chronic pain across the sample was reported as lower back pain (24.0%), with paramedics endorsing the highest prevalence of lower back pain across PSP categories (28.9%).

### Intensity and duration of chronic pain

Table 3 includes the descriptive statistics and comparative results for self-reported intensity and duration of pain. Lower back was reported as the location of most severe chronic pain (intensity and duration), with paramedics sometimes reporting significantly less intense pain than other PSP categories; nevertheless, most comparisons did not indicate statistically significant differences in severity between PSP categories. Paramedics reported experiencing shoulder pain fewer days per week than RCMP and leg pain fewer days per week than municipal/provincial police; however, there were no other statistically significant differences between PSP categories based on days per week of pain. There were no statistically significant differences between any PSP categories based on duration of pain.

### Perceived cause

Table 4 includes the descriptive statistics and comparative results for self-reported perceived causes of chronic pain. There were several statistically significant differences between PSP categories for each perceived cause. A substantial proportion (40.2%) of PSP members indicated that chronic pain was from injury related to active duty, with the highest prevalence for RCMP and paramedics (both over 50%). The next highest perceived cause was injury not related to work (16.2%), with the highest prevalence for call center operators/dispatchers (27.5%). Similarly, call center operators/dispatchers reported that a substantial proportion of chronic pain was related to non-injury-related disease (23.1%).

## Discussion

The current study provides the first Canadian chronic pain prevalence estimates including location and severity for various PSP categories. The results suggest that approximately 40.2% of PSP report current chronic pain, present more days than not for at least 3 months, which cannot be directly statistically compared to the general population estimates of approximately 25%^4–6^ but does appear substantially higher. The focus on *current* chronic pain may provide conservative estimates, and an assessment of chronic pain over the past 12 months or over a lifetime may be even higher. The largest proportion of PSP with chronic pain reported having lower back pain, which occurred on average more than 5 days per week. Importantly, almost half of all PSP participants reported that their chronic pain was related to an injury at work. Paramedics and RCMP were more likely to attribute their chronic pain to active duty–related injuries than were most other PSP groups.

The relatively high reported prevalence of chronic pain may be partially explained by the physical demands of PSP work. PSP often engage in repetitive physical activity involving substantive musculoskeletal strain coupled with extended periods of potential inactivity.^9^ As such, the opportunities for physical injury may be relatively higher than for the general population. The overall differences across PSP categories, as well as differences in physical locations of pain (e.g., lower back verses shoulder) across PSP categories, may be explained by differences in work requirements (e.g., different risks for physical task requirements). There is also evidence that increased physical activity may be beneficial for reducing the impact of chronic musculoskeletal pain, particularly among PSP.^27,38^ Accordingly, differences in opportunities to engage in regular exercise may explain some of the observed differences across PSP categories.

Beyond the physical demands, PSP are also frequently exposed to potentially traumatic events.^11,12^ There is an important link between mental disorders,^15,39,40^ particularly posttraumatic stress disorder,^13,14,^^16,17^ and chronic pain in several other populations; as such, the relatively high reports of chronic pain in PSP may be closely linked to the potentially traumatic nature of their work.^29^ More specifically, psychological and physiological vulnerabilities, possibly genetically influenced, may predispose people to develop both mental disorders and chronic pain when exposed to certain environmental conditions (e.g., an event that is both traumatic and painful).^15^ Accordingly, future research should investigate the comorbidity between chronic pain and mental disorders in PSP samples, ideally with prospective studies to assess relative causal influences and associated mechanisms (e.g., individual differences in responses to arousal, selective attention to the threat, autonomic nervous system dysregulation, endogenous opioid dysregulation^15^).

There were relatively few significant differences across PSP categories for self-reported intensity and duration of chronic pain. Nevertheless, across all PSP, lower back pain was reported as the most intense pain, whereas hand pain was reported as the least intense. The physical requirements of PSP work may mean that hand pain systematically excludes PSP from active service, whereas there may be possibilities for options to work around lower back pain. Lower back pain was reported as occurring most consistently in a given week, whereas headache pain occurred least frequently.

The most frequent attribution of cause for PSP chronic pain across categories was an injury related to active duty. Dispatchers stand out as a notable exception because significantly fewer attributed their chronic pain to an injury related to active duty. Nevertheless, RCMP and paramedics were more likely to attribute their chronic pain to an injury related to active duty than call center operators/dispatchers, corrections workers, firefighters, and municipal/provincial police. The differences may be explained, in part, by differences in PSP work requirements as well as differences in available resources and practical support for post-injury recovery. Whether and how such considerations interact with availability of sufficient health coverage (e.g., direct health care costs, compensation for lost time) remains important but impossible to clarify with the currently available data. In any case, additional research into the differences may produce opportunities to mitigate pain experiences for all PSP.

### Limitations

The large, diverse Canadian PSP sample is an important strength of the current study, as is the use of items based on established research^31–36^; however, there are several limitations that provide important directions for future research. First, participants were anonymous and self-selected, which means that despite proportional demographic representativeness, the results may not be generalizable. Related, the sampling method makes knowing the actual response rate and reasons for attrition impossible. Second, the current study focused exclusively on active duty, which may provide conservative estimates given that the data did not include those on disability. Third, the reliability and validity of web-based self-reports remains ambiguous^41^; nevertheless, the high frequencies and relatively large sample appear to justify research with more robust assessments (e.g., interviews; random sampling). Fourth, the PSP category groupings were based on previous research^29,42,43^ but could not account for potentially important differences within some categories (e.g., paramedics verses emergency medical technicians). Fifth, future research should simultaneously assess the interactive effects of mental health and chronic pain, given the substantial evidence of comorbidity^44–47^ and potentially increased risk from mutually maintaining factors.^13,48–53^

## Conclusions

The current results suggest that many PSP (i.e., 40.2%) may be experiencing chronic pain. Among those, approximately 40.2% attribute pain to an injury related to active duty and 9.6% attribute pain to an injury related to work other than active duty, which emphasizes the need for prevention efforts and, when appropriate, early interventions. There were also significant differences between PSP categories that warrant further investigation to promote physical and mental wellness. Given the overlap between trauma, mental disorders, and chronic pain, additional related research appears warranted to support calls for a national action plan in support of PSP health, both mental and physical.^8,54^
